# *Klebsiella grimontii*, a New Species Acquired Carbapenem Resistance

**DOI:** 10.3389/fmicb.2018.02170

**Published:** 2018-09-11

**Authors:** Lu Liu, Yu Feng, Yiyi Hu, Mei Kang, Yi Xie, Zhiyong Zong

**Affiliations:** ^1^Center of Infectious Diseases, West China Hospital, Sichuan University, Chengdu, China; ^2^Division of Infectious Diseases, State Key Laboratory of Biotherapy, Chengdu, China; ^3^Laboratory of Clinical Microbiology, Department of Laboratory Medicine, West China Hospital, Sichuan University, Chengdu, China; ^4^Department of Infection Control, West China Hospital, Sichuan University, Chengdu, China; ^5^Center for Pathogen Research, West China Hospital, Sichuan University, Chengdu, China

**Keywords:** carbapenemase, KPC-2, *Klebsiella*, *Klebsiella grimontii*, *Klebsiella oxytoca*

## Abstract

*Klebsiella grimontii* is a newly identified species closely related to *Klebsiella oxytoca*, but carbapenem resistance was not identified in the species before. We found a carbapenem-resistant *K. oxytoca*-like clinical strain, WCHKG020121. The strain was subjected to whole genome sequencing using Illumina HiSeq X10. The precise species identification was established based on average nucleotide identity (ANI) and *in silico* DNA–DNA hybridization (isDDH) between strain WCHKG020121 and type strains of *Klebsiella* species. Antimicrobial resistance genes were identified from the genome sequence. The sequence of the *bla*_KPC-2_-carrying plasmid was completed using PCR and Sanger sequencing. Conjugation experiments were performed to obtain the plasmid carrying *bla*_KPC-2_. All *K. grimontii* genomes were retrieved from GenBank and were analyzed for antimicrobial resistance genes. Strain WCHKG020121 was resistant to imipenem and meropenem (MIC for both, 32 μg/ml) but was susceptible to colistin (1 μg/ml). Strain WCHKG020121 was initially identified as *K. oxytoca* using Vitek II but it actually belongs to *K. grimontii* as it had a 98.81% ANI and 83.4% isDDH value with *K. grimontii* type strain. Strain WCHKG020121 had *bla*_KPC-2_; by contrast, none of other *K. grimontii* genomes carry any known carbapenemase genes. *bla*_KPC-2_ was carried by a 95,734-bp plasmid, designated pKPC2_020121, which contained two different FII(Y) replicons. pKPC2_020121 was closest (93% coverage, 99% identity) to *bla*_KPC-2_-carrying plasmids from *Enterobacter hormaechei* recovered in 2014 at the same hospital. pKPC2_020121 was not self-transmissible, which could be explained by the absence of a conjugation essential gene, *traY*. In conclusion, we reported the first *K. grimontii* strain that produced the KPC carbapenemase. Carbapenem resistant *K. grimontii* may represent a new threat.

## Introduction

*Klebsiella grimontii* is a newly identified species of the *Klebsiella* genus within the *Enterobacteriaceae* family (Passet and Brisse, 2018). *K. grimontii* is closely related to *Klebsiella oxytoca* and has been previously known as the ko6 phylogenetic group of *K. oxytoca* (Fevre et al., 2005; Passet and Brisse, 2018). Like *K. oxytoca*, a relatively common human pathogen (Herzog et al., 2014), *K. grimontii* is also associated with human infections such as bacteremia and soft tissue infection and has been found in France, Germany, and South Africa (Passet and Brisse, 2018). Carbapenems, such as imipenem and meropenem, are the main choice to treat severe infections caused by the *Enterobacteriaceae*, but carbapenem-resistant *Enterobacteriaceae* has emerged as a major threat for human health (Holt et al., 2015). Carbapenem-resistant *Klebsiella* spp., in particular *Klebsiella pneumoniae*, has been found worldwide and *bla*_KPC_ is a major determinant conferring carbapenem resistance (Tzouvelekis et al., 2012). The international dissemination of *bla*_KPC_ is largely mediated by ST258 *K. pneumoniae* (Munoz-Price et al., 2013), while in China ST11 is the major type of carbapenem-resistant *Klebsiella* (Qi et al., 2011). The plasmids carrying *bla*_KPC_ remain largely unexplored but IncF plasmids may act as a major vehicle mediating the dissemination of *bla*_KPC_ (Chmelnitsky et al., 2014). However, carbapenem-resistant *K. grimontii* has not been reported before. We have found and characterized a carbapenem-resistant *K. grimontii* clinical strain, which is reported here.

## Materials and Methods

### Strain and *in vitro* Susceptibility

Strain WCHKG020121 was recovered from a human sputum sample in 2017 in China. Initial species identification was performed using Vitek II (bioMérieux, Marcy-l’Étoile, France). MICs of amikacin, aztreonam, aztreonam/avibactam, ceftazidime, ceftazidime/avibactam, ciprofloxacin, colistin, imipenem, meropenem, piperacillin/tazobactam, tigecycline, and trimethoprim/sulfamethoxazole were determined using the broth microdilution method of the Clinical and Laboratory Standards Institute (Clinical and Laboratory Standards Institute [CLSI], 2017). As there are no breakpoints of colistin and tigecycline from CLSI, those defined by EUCAST^1^ were applied. As this study was to characterize the bacterial strain and ethical approval was not required according to the Ethical Committee of West China Hospital. No patient information was included in this study.

### Whole Genome Sequencing and Analysis

Genomic DNA of strain WCHKG020121 was prepared using the QIAamp DNA mini kit (Qiagen, Hilden, Germany) and was subjected to whole genome sequencing using the HiSeq X10 platform (Illumina, San Diego, CA, United States). Reads were trimmed using Trimmomatic (Bolger et al., 2014) and were then assembled to contigs using the SPAdes program v3.12.0 (Bankevich et al., 2012) with careful mode turned on. Annotation of the genomic sequence was carried out using the Prokka program v1.12 (Seemann, 2014). The precise species identification was established based on average nucleotide identity (ANI) and *in silico* DNA-DNA hybridization (isDDH) between strain WCHKG020121 and type strains of *Klebsiella* species (**Table 1**) using JSpeciesWS (Richter et al., 2016) and GGDC (formula 2) (Meier-Kolthoff et al., 2013), respectively. Antimicrobial resistance genes were identified from the genome sequence using the ABRicate program^2^ to query the ResFinder database^3^. The sequence of the *bla*_KPC-2_-carrying plasmid, a carbapenemase-encoding gene, was completed using PCR and Sanger sequencing to close gaps between contigs. Plasmid replicon types were determined using the PlasmidFinder tool at https://cge.cbs.dtu.dk/services/PlasmidFinder/ and the allele types of IncF plasmids were assigned using the IncF replicon typing tool (Villa et al., 2010).

**Table 1:** Average nucleotide identity (ANI) and isDDH values between strain WCHKG020121 and the type strains of *Klebsiella* species.

Species	Strain	Accession no.	ANI (%)
*K. aerogenes*	KCTC2190	CP002824	82.08
*K. grimontii*	06D021	FZTC01000000	98.81
*K. michiganensis*	H1g	AYMI01000000	93.22
*K. oxytoca*	NBRC105695	BCZK01000000	90.95
*K. pneumoniae* subsp. *ozaenae*	ATCC 11296	CDJH01000000	82.62
*K. pneumoniae* subsp. *pneumoniae*	ATCC 13883	JOOW01000000	82.54
*K. pneumoniae* subsp. *rhinoscleromatis*	ATCC 13884	CDOT01000000	82.74
*K. quasipneumoniae* subsp. *quasipneumoniae*	01A030	CCDF01000000	83.22
*K. quasipneumoniae* subsp. *similipneumoniae*	07A044	CBZR010000000	82.89
*K. quasivariicola*	KPN1705	CP022823	82.47
*K. variicola*	DSM15968	CP010523	82.58
*R. planticola*	B43	BADH01000000	77.48
*R. ornithinolytica*	ATCC 31898	NC_021066	83.09
*R. terrigena*	ATCC 33257	LANE01000000	83.65


#### Nucleotide Sequence Accession Number

The draft whole-genome sequence of strain WCHKG020121 and the complete sequence of pKPC2_020121 have been deposited into GenBank under the accession no. QBDY00000000 and MH192342, respectively.

### Analysis on *K. grimontii* Genomes Available in GenBank

*Klebsiella grimontii* carries the chromosomally based *bla*_OXY-6_ β-lactamase gene, which is the marker of *K. grimontii* (Passet and Brisse, 2018). All genomes containing *bla*_OXY-6_ were therefore retrieved from GenBank (accessed by May 1, 2018, **Table 2**) and were subjected to the precise species identification using ANI with the type strain of *K. grimontii* as described above. Antimicrobial resistance genes were identified using ResFinder.

**Table 2:** Genomes and antimicrobial resistance genes of *K. grimontii* strains.

Strain	GenBank accession no.	Species assignation in GenBank record	ANI (%) with 06D021	Location	Host	Year of collection	*bla*_OXY-6_ variant	Carbapenemase gene	Other resistance genes^1^
WCHKG020121	QBDY00000000	*K. grimontii*	98.81	China	Human	2017	*bla*_OXY-6-4_	*bla*_KPC-2_	β-lactams (*bla*_LAP-2_), quinolones (*qnrS1*), rifampicin (*catA*), and tetracycline [*tet(A)*].
06D021	FZTC00000000	*K. grimontii*	–	France	Human	1997	*bla*_OXY-6-1_	–	–
M5al	CP020657	*Klebsiella* sp.	99.41	China	NA	NA	*bla*_OXY-6-4_	–	–
JKo3	AP014951	*K. oxytoca*	99.38	Japan	NA	NA	*bla*_OXY-6-2_	–	–
371_KOXY	JVJS00000000	*K. michiganensis*	99.09	United States	Human	NA	*bla*_OXY-6-4_	–	–
375_KOXY	JVJO00000000	*K. michiganensis*	99.05	United States	Human	NA	*bla*_OXY-6-4_	–	–
397_KOXY	JVIT00000000	*K. michiganensis*	99.06	United States	Human	NA	*bla*_OXY-6-4_	–	–
409_KOXY	JVIH00000000	*K. michiganensis*	99.07	United States	Human	NA	*bla*_OXY-6-4_	–	–


*^*1*^All *K. grimontii* strains have *fosA* (mediating resistance to fosfomycin) and *oqxA*/*oqxB* (mediating resistance to quinolones).*

### Mating

Conjugation experiments were carried out in blood heart infusion broth (Oxoid, Hampshire, United Kingdom) and on nitrocellulose filters (GE Life Science, Pittsburgh, PA, United States) at both 30 and 37° as described previously (Coque et al., 2002; Novais et al., 2006; Valenzuela et al., 2007). An azide-resistant *Escherichia coli* strain J53 was used as the recipient. For the broth method, the donor and recipient were mixed at a ratio of 1:10 and the mixture was incubated overnight. For the filter method, the donor and recipient were mixed at a ratio of 1:1 and the mixture was incubated for 4 h. Transconjugants were then selected on LB agar plates containing 4 μg/ml meropenem and 150 μg/ml azide.

## Results and Discussion

Strain WCHKG020121 was resistant to aztreonam (MIC, 512 μg/ml), ceftazidime (64 μg/ml), imipenem (32 μg/ml), meropenem (32 μg/ml), piperacillin/tazobactam (512/4 μg/ml) and tigecycline (4 μg/ml), intermediate to ciprofloxacin (2 μg/ml) and susceptible to amikacin (1 μg/ml), aztreonam/avibactam (<0.125/4 μg/ml), ceftazidime/avibactam (0.25/4 μg/ml), colistin (1 μg/ml), and trimethoprim/sulfamethoxazole (<0.5/9.5 μg/ml).

Whole genome sequencing generated 6,957,050 reads and 2.09 Gb clean bases, which were *de novo* assembled to 127 contigs (108 > 1,000 bp; *N_50_* 107,740 bp). The draft genome of strain WCHKG020121 was 6.28 Mb with a 55.56% GC content. Strain WCHKG020121 was initially identified as *K. oxytoca* using Vitek II. However, strain WCHKG020121 had 98.81% ANI value with strain 06D021^T^, the type strain of *K. grimontii* (Passet and Brisse, 2018), while the ANI values between strain WCHKG020121 and types strains of other *Klebsiella* spp. were 82.54 to 93.22% (**Table 1**). A =95–96% ANI value (Richter and Rossello-Mora, 2009) is commonly used to define a bacterial species. The isDDH value between strain WCHKG020121 and the type strain of *K. grimontii* was 83.4%, which is above the 70% cutoff to define a bacterial species. Therefore, strain WCHKG020121 actually belongs to *K. grimontii*.

The strain had nine antimicrobial resistance genes mediating resistance to β-lactams (*bla*_KPC-2_, *bla*_LAP-2_, *bla*_OXY-6-4_), fosfomycin (*fosA*), quinolones (*oqxA, oqxB, qnrS1*), rifampicin (*catA*), and tetracycline [*tet(A)*]. There are seven *Klebsiella* genomes containing *bla*_OXY-6_ available in the GenBank(**Table 2**). Although these strains were commonly reported as *K. oxytoca* or *Klebsiella michiganensis* in their records in GenBank, they had >99% ANI values with *K. grimontii* type strain 06D021 (**Table 2**), which clearly suggests that the strains actually belonged to *K. grimontii*. None of these *K. grimontii* strains carried any known carbapenemase genes (**Table 2**), although the susceptibility data of carbapenems against these strains were not available. To our knowledge, this is the first report of a carbapenemase-producing *K. grimontii*, which expands the species spectrum of carbapenem-resistant *Enterobacteriaceae*. Curiously, all *K. grimontii* genomes analyzed contained *fosA, oqxA*, and *oqxB*, being needed more analyses to verify if they could be intrinsic of this species. In contrast, none of other antimicrobial resistance genes (*bla*_LAP-2_, *qnrS1, catA*, and [*tet(A)*]) seen in strain WCHKG020121 was present in other *K. grimontii* genomes, suggesting that these genes were acquired by strain WCHKG020121.

**FIGURE 1:**
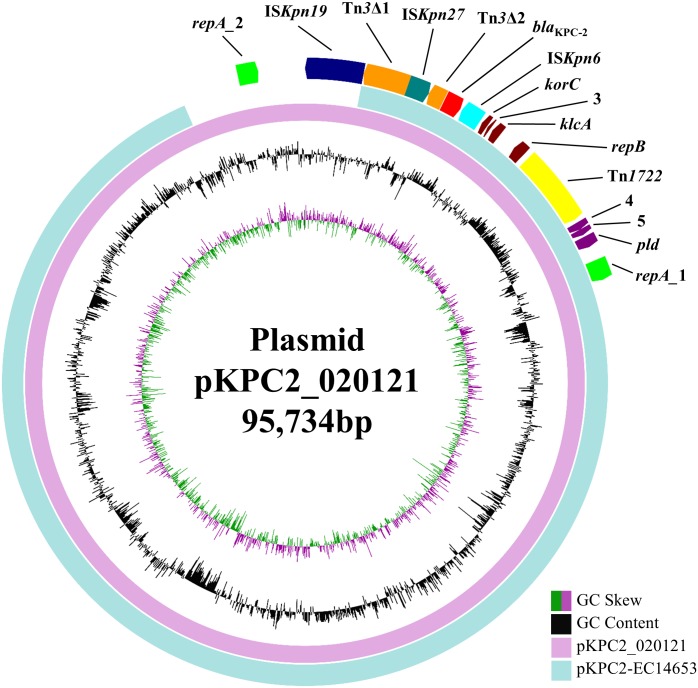
pKPC2_020121 vs. pKPC2-EC14653. pKPC2_020121 (in pink) is aligned with pKPC2-EC14653 (GenBank accession no. KP868646; in light blue), a plasmid from *E. hormaechei* recovered in 2014 at our hospital, using BRIG (Alikhan et al., 2011). pKPC2_020121 has two FII(Y) replicon, while pKPC2-EC14653 has one. GC content and GC skew are indicated. The genetic context of *bla*_KPC-2_ is shown in **Figure 2**. The annotation of the genetic components were added manually using the Microsoft PowerPoint program.

*bla*_KPC-2_ was carried by a 95,734-bp plasmid, which is designated pKPC2_020121 here. pKPC2_020121 contained two different FII(Y) replicons, in which the 227-bp allele used by the PlasmidFinder tool to define FII was 91.63% identical between the two replicons. pKPC2_020121 was closest (93% coverage, 99% identity) to 88,213-bp *bla*_KPC-2_-carrying plasmids pKPC2-EC14653, pKPC2_EClY2402 and pKPC2_EClY2403 (GenBank accession no. KP868646, KY399972, and KY399973; **Figure 1**). The three plasmids were identical except several nucleotide differences and were found in three *Enterobacter hormaechei* isolates, which were recovered in 2014 at our hospital and were likely of a common strain (Yang et al., 2018). Nonetheless, pKPC2-EC14653/pKPC2_EClY2402/pKPC2_EClY2403 had only one FII(Y) replicon (**Figure 1**). A 7-kb region containing the additional FII(Y) replicon on pKPC2_020121 were absent from the three plasmids; otherwise, pKPC2_020121 were almost identical to pKPC2-EC14653/pKPC2_EClY2402/pKPC2_EClY2403 with only a few nucleotide mutations or insertions/deletions. It is likely that pKPC2_020121 and pKPC2-EC14653/pKPC2_EClY2402/pKPC2_EClY2403 had originated from a common plasmid to mediate inter-species transfer of *bla*_KPC-2_ at our hospital and pKPC2_020121 might have acquired the additional FII(Y) replicon during its transfer. The presence of an additional replicon may facilitate the host plasmid to adapt to different strains of different species.

Transconjugants were not obtained despite repeated attempts. We found that the relaxosome protein-encoding gene *traY*, which is an essential component of the conjugation module, was absent from pKPC2_020121. This could explain that pKPC2_020121 was not self-transmissible. Nonetheless, in our previous study, we have found that pKPC2-EC14653 could be transferred in the presence of a self-transmissible FII plasmid (Wu et al., 2015) suggesting that pKPC2_020121 may also utilize this mechanism to realize its transmission. Plasmid replicon typing revealed that strain WCHKG020121 had an FII(K) and an FIB(K) replicon in addition to pKPC2_020121, suggesting that there is one more FII plasmid in the strain.

**FIGURE 2:**
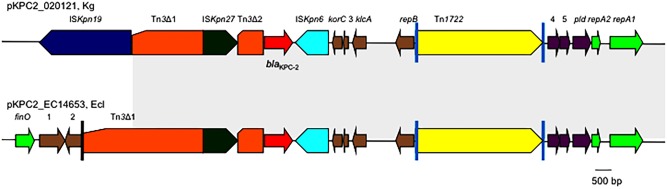
The genetic context of *bla*_KPC-2_ on pKPC2_020121 and pKPC2-EC14653. The genetic context of *bla*_KPC-2_ on pKPC2_020121 is compared with that on pKPC2-EC14653 with the identical region highlighted in gray. Poles in black are the inverted repeats (IR) of Tn*3*, while poles in cyan are the IR of Tn*1722*. orfs without known function are indicated by numbers 1–5. This figure was drawn manually using the Microsoft PowerPoint program.

The genetic context of *bla*_KPC-2_ on pKPC2_020121 was almost identical to those on pKPC2-EC14653/pKPC2_EClY2402/pKPC2_EClY2403 (**Figure 2**). The only difference is that the Tn*3* transposon upstream of *bla*_KPC-2_ was truncated by IS*Kpn19*, resulting in the absence of the Tn*3* inverted repeat (IR) from pKPC2_020121.

## Conclusion

We reported a carbapenem-resistant strain of the newly recognized species *K. grimontii*. Carbapenem resistance was due to *bla*_KPC-2_, which was carried by a plasmid containing two FII(Y) replicons. The *bla*_KPC-2_-carrying plasmid had circulated in different species at the hospital for several years.

## Author Contributions

ZZ designed the study. LL, YH, YX, and MK performed the experiments. LL, YF, and ZZ analyzed and interpreted the data. ZZ wrote the manuscript.

## Conflict of Interest Statement

The authors declare that the research was conducted in the absence of any commercial or financial relationships that could be construed as a potential conflict of interest.

## Footnotes

1 http://www.eucast.org/

2 https://github.com/tseemann/abricate

3 https://cge.cbs.dtu.dk/services/ResFinder/
